# MRSA-related knowledge, attitudes, and practices among clinical dental students: a cross-sectional study with implications for infection prevention and control education and training

**DOI:** 10.3389/froh.2026.1928942

**Published:** 2026-08-31

**Authors:** Thikrayat Badrasawi, Farah Tuqan, Leen Abdul Fattah, Layan Odeh, Aya Obeid, Aysha Bashar Qanaze, Aya Abu Aqab

**Affiliations:** 1 Basic Dental Sciences Department, Faculty of Dentistry, An-Najah National University, Nablus, Palestine; 2 Public Health PhD Program, Faculty of Graduate Studies, Al-Quds University, Jerusalem, Palestine; 3 Undergraduate Dental Student, Faculty of Dentistry, An-Najah National University, Nablus, Palestine

**Keywords:** Methicillin-resistant Staphylococcus aureus (MRSA), knowledge, attitudes, practices (KAP), dental students, infection prevention and control (IPC), dental education, antimicrobial resistance

## Abstract

**Background:**

Methicillin-resistant *Staphylococcus aureus* (MRSA) is an important public health concern because of its role in healthcare-associated infections, antimicrobial resistance, and potential transmission in clinical environments. Dental students may be exposed to MRSA through patient contact, aerosols, contaminated surfaces, and routine clinical procedures. This study assessed MRSA-related knowledge, attitudes, and practices (KAP) among clinical dental students and identified predictors of infection-control practice.

**Methods:**

A cross-sectional study was conducted among clinical dental students at the Faculty of Dentistry, An-Najah National University. A structured questionnaire was used to assess sociodemographic and academic characteristics, previous MRSA-related exposure, infection prevention and control (IPC) training, and KAP domains. Knowledge, attitude, and practice scores were calculated and classified into levels. Non-parametric tests were used to assess group differences, Spearman's correlation was used to examine relationships between KAP scores, and multiple linear regression was performed to identify predictors of infection-control practice score.

**Results:**

A total of 166 valid responses were analyzed. The mean knowledge score was 8.38 ± 3.96 out of 14, while attitude and practice scores were 43.02 ± 7.40 out of 60 and 48.14 ± 7.27 out of 55, respectively. Poor knowledge was observed in 47.6% of students, whereas 80.7% reported good practice. Knowledge was positively correlated with attitude and practice (*p* < 0.001 for both). In adjusted linear regression, higher knowledge score (*p* = 0.003), more frequent IPC training (*p* < 0.001), female gender(*p* = 0.031), third- and fourth-year study level (*p* = 0.017 for both), and GPA 3.0-3.49 (*p* = 0.024) were significantly associated with higher practice scores. The model explained 39.5% of the variance in practice.

**Conclusion:**

Clinical dental students reported good infection-control practices despite gaps in MRSA-specific knowledge. Strengthening MRSA-related education and repeated IPC training may improve students' preparedness for safe clinical practice.

## 1 Introduction

Methicillin-resistant *Staphylococcus aureus* (MRSA) is an important bacterium in healthcare settings. It is a strain of *Staphylococcus aureus* resistant to beta-lactam antibiotics, including methicillin (1), and can cause a wide range of infections, such as skin, soft tissue, and surgical site infections, pneumonia, bacteremia, osteomyelitis, and endocarditis (1). This pathogen is commonly classified into hospital-acquired (HA-MRSA), which is mainly associated with healthcare settings, and community-acquired (CA-MRSA), which occurs in outpatient and community environments (2). MRSA is considered a major pathogen associated with a substantial burden of serious diseases (3), and remains a major concern in healthcare because of its ability to spread through direct contact, contaminated hands, environmental surfaces, and, in some situations, respiratory secretions. In dental settings, the risk of transmission is particularly relevant due to close contact with patients, and the contaminated hands of dental healthcare workers, frequent exposure to saliva and blood, and the generated aerosols and splashes during clinical procedures (4). In addition, dental equipment and environmental surfaces (5), such as dental chairs, light handles, syringes, and spittoons may serve as potential reservoirs if infection control measures are inadequate (6).

MRSA was first identified in England in the 1960s, shortly after methicillin was introduced for clinical use and by the late 1970s it had emerged as a major cause of healthcare-associated infections in American hospitals (7). MRSA contributes to 50−70% of hospital-acquired *S. aureus* infections (8), this high proportion highlights its major role in hospital acquired infections and demonstrates the challenge it poses for infection prevention and control (IPC) and antimicrobial treatment. The clinical impact of MRSA is also substantial, the CDC has reported approximately 70,000 invasive MRSA infections and 9,000 related deaths annually in the United States (9) 11, while MRSA bacteremia in England was associated with case-fatality rates of 20% within 7 days and 38% within 30 days, underlining the seriousness of invasive MRSA bloodstream infections (10). Although prevalence varies geographically, high resistance rates have also been reported, in Asia, specifically Korea, methicillin resistance exceeded 50% among *S. aureus* isolates and around 13,000 invasive MRSA cases were estimated in 2010 (11, 12), these data emphasizes the continuous need of IPC programs to decrease the burden and the increased antibiotic resistance of MRSA. In addition to its medical effects, MRSA infections imposes an economic burden on healthcare systems, by prolonging hospital stays, and necessitating the use of broad spectrum and expensive antibiotics, increasing care costs and complexity (13).

In dentistry, MRSA is of particular importance due to the close patient contact, exposure to saliva and blood, and many dental procedures being aerosol-generating, which may facilitate the spread of microorganisms, including MRSA (14–16). Environmental contamination in dental settings has been documented in previous research. In one cross-sectional study, 354 samples were collected from six surfaces of dental equipment before and after patient consultations. The highest contamination with MRSA was found on the dental chair armrest (29.7%), followed by the headboard (27%) and the dental spittoon (24.3%). Similarly, a Saudi study from King Saud University screened MRSA contamination on 10 surfaces in five dental specialty clinics before and after treatment of patients. The study found increased MRSA contamination after patient visits on similar frequently touched clinical surfaces, with the highest contamination reported on paper dental records in the Oral Medicine clinic, followed by high counts on the x-ray viewer (17). These findings supports that dental clinic surfaces may serve as reservoirs for cross-contamination and possible transmission (5). MRSA carriage among Dental health care personnel (DHCP) is a particularly an important area of concern. A Kuwaiti study reported notable MRSA carriage in dental settings, carriage rates on patients hands of 9.8%, 6.6% among nurses, and 5% among dentists, The anterior nares are recognized as the most common site of MRSA colonization, the same study reported nasal colonization of 9.7% in dentists and 11.1%in patients (15), which raises concern about possible transmission from dental personnel themselves through direct or indirect contact with contaminated hands and surfaces. Similarly, another study found an 18.5% prevalence of nasal MRSA carriage among dental students, with higher rates among postgraduates than undergraduates, suggesting that increased clinical exposure may contribute to MRSA colonization risk (14).

These findings from previous international studies has highlighted the potential role of dental settings in MRSA transmission, and the role of colonization in facilitating cross-transmission, community spread (18), and the continuous expanding antimicrobial-resistant (19), therefore, dental students are a key group to investigate (16), because they are in the process of developing both theoretical knowledge and clinical infection control skills. Evaluating their knowledge and practices regarding MRSA can help identify gaps in education and support improvements in IPC training (8). In Palestine, a previous study assessed MRSA-related KAP, and nasal carriage among dental students, with emphasis on microbiological carriage and associated colonization factors (20). However, further local evidence is still needed from other Palestinian dental education settings, particularly regarding the educational and behavioral factors associated with infection-control practice. Therefore, the present study was conducted among clinical dental students at An-Najah National University to assess MRSA-related knowledge, attitudes, and infection-control practices and to identify predictors of better MRSA-IPC practice, that may support the strengthening of MRSA-related education and repeated IPC training within dental curricula. This may also provide an indirect reflection of broader standard precaution training and IPC practice in dental clinical settings. This approach may also help reflect students' broader readiness to apply standard precautions during clinical dental care.

## 2 Methodology

### 2.1 Study design and population

This study was designed as a cross-sectional questionnaire-based study conducted to assess participants' knowledge, attitudes, and infection control practices regarding MRSA. The target population consisted of approximately 600 clinical students at the faculty of dentistry, at An Najah National University in Palestine, including third-, fourth-, and fifth-year students, with clinical exposure beginning in the third year. Eligible students who were willing to participate were invited to complete the questionnaire. Subjects who were not part of the target population, declined to participate, or submitted incomplete questionnaires were excluded from the analysis. The required sample size was calculated using Cochran's formula for a single population proportion: *n* = Z^2^p(1−p)/d^2^, where Z = 1.96 for a 95% confidence level, 5% margin of error, 50% expected proportion. Finite population correction was applied using the formula: *n* = n₀/[1 + (n₀−1)/N], giving a required sample size of approximately 234 students. A total 166 valid responses were included in the final analysis. A non-probability convenience sampling technique was used, and 166 valid responses were included in the final analysis. Due to voluntary participation, these responses represented 70.9% of the calculated required sample size, and 27.7% of the estimated eligible student population.

### 2.2 Measurement tools and data collection

Data were collected electronically between April 1 and June 1, 2026, using a structured self-administered questionnaire. The questionnaire link was distributed to eligible students through institutional email and WhatsApp groups. Reminder messages were sent through the same channels to improve the response rate. Duplicate submissions were minimized by restricting the questionnaire to one response per institutional email account. The questionnaire consisted of four main sections. The first section included socio-demographic information. The second section assessed participants' knowledge about MRSA through 14 items covering the general concepts about MRSA, common modes of transmission, and effective sterilization and disinfection methods. Knowledge items were scored as 1 for correct answers and 0 for incorrect or “I do not know” responses. The third section assessed participants' attitudes toward MRSA prevention and infection control using 12 items measured on a five-point Likert scale, ranging from strongly agree to strongly disagree. These items explored perceptions of MRSA risk, the importance of infection control, and the perceived responsibility of dental students in preventing transmission. The fourth section evaluated self-reported infection control practices using 11 items. These items focused on compliance with hand hygiene, use of personal protective equipment, use of high-volume evacuators during aerosol-generating procedures, and adherence to surface disinfection protocols. Negatively worded items, were reverse-coded before analysis. Knowledge and practice were classified as poor (< 60%), moderate (60−79%), and good (≥ 80%). Attitude was classified as weak (< 60%), moderate (60−79%), and positive (≥ 80%). These cutoffs were based on the Bloom's cut-off approach commonly used in KAP studies (21, 22)

This tool was developed for the present study based on previous MRSA-related KAP studies and infection-control literature, then adapted to fit the clinical dental student context. Prior to the primary data collection, a pilot study was conducted with 10 dental students who were not included in the main study to evaluate the internal consistency, clinical relevance and clarity of the research instrument. The questionnaire's reliability was assessed using Cronbach's alpha for the attitude and practice sections, while the Kuder-Richardson Formula 20 (KR-20) was applied to the dichotomously scored knowledge items. The questionnaire demonstrated acceptable to good internal consistency with KR-20 = 0.712, Cronbach's alpha values of 0.824 and 0.923 for the attitude and practice domains, respectively.

### 2.3 Data analysis

Data were analyzed with IBM SPSS statistics 26. Descriptive statistics were used to summarize participants' demographic characteristics and questionnaire responses. Categorical variables were presented as frequencies and percentages, while continuous variables were presented as means and standard deviations or medians and interquartile ranges, depending on the data distribution. Normality of continuous KAP scores was assessed using the Shapiro–Wilk test and visual inspection of histograms and Q-Q plots, since the data was not normally distributed, non-parametric inferential tests were used. The Mann–Whitney U test was used to compare KAP scores between two-category variables, while the Kruskal–Wallis test was used for variables with more than two categories. When Kruskal–Wallis tests showed significant differences, *post-hoc* pairwise comparisons performed to identify the source of differences between groups. Spearman's correlation was used to assess the relationships between knowledge, attitude, and practice scores. Multiple linear regression was performed to identify predictors of good infection-control practice, Unstandardized regression coefficients with 95% confidence intervals were reported. A *p*-value of less than 0.05 was considered statistically significant.

### 2.4 Ethical considerations

The study received ethical approval from the Institutional Review Board (IRB) at An-Najah National University with reference number [Dent.Mar.2026/37]. Informed consent was obtained electronically through the first page of the online questionnaire before participation. This page explained the purpose of the study, voluntary nature of participation, confidentiality of responses, and the participants' right to withdraw at any time without any consequences. The collected information was anonymized and coded securely with limited access to the main investigator and reported in aggregate form only.

## 3 Results

A total of 166 valid responses were included in the final analysis. The characteristics of the participants are presented in Table 1. The sample was mainly composed of female students, most participants reported occasional patient interaction during clinical training, and the academic curriculum was the most common reported source of MRSA-related knowledge.

**Table 1:** Sociodemographic and academic characteristics of participants.

Variable	Category	*n*	%
Age	18–20	11	6.6
	21–23	148	89.2
	24–26	5	3.0
	>26	2	1.2
Gender	Female	122	73.5
	Male	44	26.5
Year of study	Third year	44	26.5
	Fourth year	47	28.3
	Fifth year	75	45.2
Patient interaction	Rarely	50	30.1
	Occasionally	97	58.4
	Frequently	19	11.4
GPA range	Below 2.5	6	3.6
	2.5–2.99	79	47.6
	3.0–3.49	53	31.9
	3.5–4.0	28	16.9
Previous awareness of MRSA	Yes	120	72.3
	No	46	27.7
Previous educational exposure to *S. aureus*/MRSA	Yes	140	84.3
	No	26	15.7
Main source of knowledge/ educational exposure	Course lecture/curriculum	97	58.4
	General knowledge	29	17.5
	Scientific articles/journals	16	9.6
	Internet/social media	15	9.0
	Personal assumption/no specific source	8	4.8
	Other	1	0.6

Percentages may not sum to 100% due to rounding.

Table 2 presents the overall KAP scores and their level classifications. Knowledge showed the lowest mean percentage score with almost half of the students were classified to have poor scores, whereas most participants reported to have good self-reported practice.

**Table 2:** Classification of knowledge, attitude, and practice levels.

Domain	Subcategory	*n* (%)	Items	Possible range	Mean ± SD	Median	IQR	Mean %
Knowledge	Overall score	166	14	0–14	8.38 ± 3.96	9	6–12	59.9
	Poor	79 (47.6)						
	Moderate	45 (27.1)						
	Good	42 (25.3)						
Attitude	Overall score	166	12	12–60	43.02 ± 7.40	44	38–48	71.7
	Weak/negative	19 (11.4)						
	Moderate	103 (62.1)						
	Positive	44 (26.5)						
Practice	Overall score	166	11	11–55	48.14 ± 7.27	50	45–54	87.5
	Poor	2 (1.2)						
	Moderate	30 (18.1)						
	Good	134 (80.7)						

Item-level analysis of the knowledge, attitude, and practice domains showed variation across specific MRSA-related concepts and infection-control behaviors. Knowledge gaps were more evident in specific rather general MRSA-related concepts, while self-reported adherence was higher for routine infection-control practices. Full item-level results are presented in Supplementary Tables 1−3. Internal consistency was reassessed using the full analytic sample, the questionnaire demonstrated acceptable internal consistency, with KR-20 = 0.874 for the knowledge domain and Cronbach's alpha values of 0.802 and 0.884 for the attitude and practice domains, respectively.

The association between participant characteristics and KAP scores showed significant association with year of study was significantly associated with all KAP domains. Patient interaction frequency and GPA were significantly associated with practice scores, while previous MRSA awareness, source of knowledge, and IPC training frequency showed significant associations with some KAP domains presented in Table 3.

**Table 3:** Association between participant characteristics and KAP scores.

Grouping variable	Category (n)	Knowledge median (IQR)	Knowledge *p*-value	Attitude median (IQR)	Attitude *p*-value	Practice median (IQR)	Practice *p*-value
Gender	Female (122)	9 (6–12)	0.157	45 (39–49)	**0.037**	51 (45–54)	0.099
	Male (44)	8 (6–10)		42.5 (37–46)		48 (42–54)	
Year of study	Third year (44)	11 (10–14)	**<0.001**	46 (44–49)	**<0.001**	54 (51–55)	**<0.001**
	Fourth year (47)	8 (5–12)		46 (40–50)		51 (46–54)	
	Fifth year (75)	7 (5–10)		42 (36–46)		46 (41–50)	
Patient interaction frequency	Frequently (>7 patients/week) (19)	9 (4–10)	0.058	39 (36–48)	0.084	48 (45–54)	**0.001**
	Occasionally (3–7 patients/week) (97)	8 (6–11)		44 (38–47)		49 (44–53)	
	Rarely (<2 patients/week) (50)	10 (7–13)		46 (42–49)		54 (49–55)	
GPA range	Below 2.5 (6)	10.5 (4–12)	0.867	44 (42–47)	0.266	54 (51–55)	**0.030**
	2.5–2.99 (79)	8 (5–11)		43 (36–48)		50 (44–54)	
	3.0–3.49 (53)	9 (6–12)		44 (38–48)		51 (48–55)	
	3.5–4.0 (28)	9 (7–10)		46 (44–48)		47 (44–51)	
Previous MRSA awareness	No (46)	5 (1–7)	**<0.001**	37.5 (36–43)	**<0.001**	46.5 (37–52)	**0.001**
	Yes (120)	10 (8–12)		46 (42–49)		51 (46–55)	
Received S. aureus/MRSA information	No (26)	7.5 (2–10)	**0.019**	42.5 (36–47)	0.139	45.5 (38–54)	0.081
	Yes (140)	9 (6–12)		45 (38–48)		50 (46–54)	
Source of knowledge	Course lecture/curriculum (97)	10 (8–13)	**<0.001**	46 (42–49)	**<0.001**	51 (48–55)	**0.001**
	General knowledge (29)	8 (6–10)		45 (41–47)		50 (44–54)	
	Scientific articles/journals (16)	6 (4–10)		38.5 (35–45)		45.5 (38–54)	
	Internet/social media (15)	6 (4–10)		37 (35–46)		38 (34–49)	
	Personal assumption/no specific source (8)	2 (2–3)		36 (36–38)		48.5 (40–54)	
Previous IPC workshop/training frequency	Never (16)	6 (2–7)	**0.024**	42 (37–45)	0.427	45 (40–49)	**<0.001**
	Rarely (30)	10 (6–11)		45.5 (43–49)		48 (44–54)	
	Sometimes (33)	9 (5–12)		44 (37–48)		47 (36–52)	
	Often (36)	9 (6–12)		46 (37–48)		50 (46–53)	
	Always (51)	9 (7–12)		44 (38–47)		54 (51–55)	

The “Other” category was excluded from group comparison because it included only one participant.

Bold values indicate statistically significant results (*p* < 0.05).

Post-hoc pairwise comparisons were performed for variables with significant Kruskal–Wallis results. Third-year students had significantly higher knowledge scores than both fourth- and fifth-year students, and higher practice scores than fourth- and fifth-year students. For source of knowledge, curriculum-based learning was associated with higher knowledge and practice scores than internet/social media or personal understanding/no specific source. Regarding IPC training frequency, higher training exposure was associated with higher knowledge and practice scores. Detailed *post-hoc* comparisons are presented in Supplementary Table 4.

The correlations between KAP scores are shown in Table 4. Knowledge was positively correlated with both attitude and practice. The strongest correlation was observed between knowledge and attitude.

**Table 4:** Correlation between KAP scores.

Correlation pair	Spearman's rho	*p*-value	Interpretation
Knowledge and attitude	0.572	<0.001	Strong positive correlation
Knowledge and practice	0.426	<0.001	Moderate positive correlation
Attitude and practice	0.267	0.001	Weak positive correlation

Multiple linear regression was performed and female gender, third- and fourth-year study level, GPA 3.0-3.49, higher knowledge score, and more frequent IPC training were significantly associated with higher infection-control practice scores. The model explained 39.5% of the variance in infection-control practice (adjusted R^2^ = 0.395, *p* < 0.001). Table 5

**Table 5:** Multiple linear regression for predictors of infection-control practice score.

Predictor	Category/unit	B	95% CI	*p*-value
Knowledge score	Per 10% increase	1.34	0.47–2.20	**0.003**
Attitude score	Per 10% increase	0.22	−1.34–1.78	0.783
Gender	Female vs. Male	4.12	0.37–7.87	**0.031**
Year of study	Third year vs. fifth year	6.42	1.15–11.69	**0.017**
Year of study	Fourth year vs. fifth year	5.52	1.00–10.05	**0.017**
Patient interaction frequency	Occasionally vs. rarely	−2.45	−6.89–1.99	0.278
Patient interaction frequency	Frequently vs. rarely	1.70	−4.69–8.08	0.600
GPA range	Below 2.5 vs. 2.5–2.99	8.27	−3.25–19.79	0.158
GPA range	3.0–3.49 vs. 2.5–2.99	3.96	0.54–7.38	**0.024**
GPA range	3.5–4.0 vs. 2.5–2.99	0.51	−4.60–5.61	0.845
Received information about S. aureus/MRSA	Yes vs. No	4.15	−1.03–9.34	0.116
IPC training/workshop frequency	Per one-category increase	2.92	1.42–4.43	**<0.001**

CI, confidence interval. Statistical significance was set at *p* < 0.05. Model fit: R^2^ = 0.439, adjusted R^2^ = 0.395.

Bold values indicate statistically significant results (*p* < 0.05).

## 4 Discussion

The present study showed that clinical dental students generally reported good infection-control practices, although MRSA-specific knowledge remained limited in a considerable proportion of participants. Knowledge was positively correlated with both attitude and practice, and in the adjusted linear regression model, higher knowledge score and more frequent IPC training were significant predictors of better practice. These findings suggest that good IP practice is not simply a checklist behavior, but the clinical translation of knowledge reinforced through training, supervision, and educational exposure.

In the adjusted linear regression model, higher MRSA-related knowledge score was significantly associated with higher IPC practice score. Each 10% increase in knowledge score was associated with a 1.34 percentage-point increase in practice score, indicating that knowledge was not only a theoretical outcome but was translated into better self-reported clinical behavior. This finding supports the educational concept that safe clinical practice depends on an adequate cognitive foundation. According to Miller's pyramid of clinical competence, knowledge represents the base of clinical competence and progresses from “knows” to “knows how,” “shows how,” and finally “does” in real clinical practice (23). This finding is consistent with recent dental KAP study showing that students may report positive attitudes and recommended IPC practices despite insufficient knowledge, suggesting that routine compliance alone may not reflect complete educational preparedness (24). Therefore, MRSA-related knowledge should not be viewed as isolated microbiological information, but as an essential foundation for infection-control behavior in dental clinics. WHO states that IPC education for health-related students should equip students with essential knowledge, skills, and behavioral competencies for safe care, reducing healthcare-associated infections and antimicrobial resistance (25). Students who understand MRSA colonization, transmission pathways, environmental persistence, and antimicrobial resistance are more likely to appreciate the rationale behind hand hygiene, PPE use, surface disinfection, and other preventive measures. This supports the need to reinforce MRSA-related education during clinical training so that knowledge can mature into professional attitude and consistent infection-control practice.

More frequent IPC training was significantly associated with higher infection-control practice scores. IPC training frequency was represents a modifiable educational factor that may influence infection-control behavior. In this study, more frequent IPC training was significantly associated with higher infection-control practice scores, in the adjusted linear regression model, each one-category (never, rarely, sometimes, often, and always) increase in training frequency was associated with a 2.92 percentage-point increase in practice score, supporting the role of repeated training in reinforcing standard precautions and safe clinical behavior. This finding is consistent with previous evidence suggesting that repeated IPC training may help transform infection-control knowledge into consistent clinical behavior and better compliance (26, 27). Unlike theoretical knowledge alone, practical training and workshops provide students with repeated opportunities to apply infection-control principles, practice hand hygiene, use PPE correctly, perform surface disinfection, and follow safe clinical workflow. This is consistent with the WHO IPC pre-service curriculum, which emphasizes that health-related students should acquire not only IPC knowledge, but also the practical skills and behavioral competencies required for safe healthcare delivery and reduction of healthcare-associated infections and antimicrobial resistance (25). It is also supported by dental education study in showing that comprehensive infection-control training can improve students' knowledge, awareness, and IPC practices, particularly during the transition from preclinical to clinical training (28). Similarly, Antoniadou et al. found that structured educational interventions improved infection-control awareness among dental students (29), another study among Jordanian nurses reported that an online standard precautions education module improved undergraduate nursing students' knowledge and compliance with infection-control precautions (30). These findings indicate that IPC training should not be delivered as a single introductory session, but should be repeatedly reinforced throughout clinical dental education through workshops, simulation, direct supervision, and competency-based assessment (28).

Year of study was significantly associated with infection-control practice score. Third and fourth year students had higher practice scores than fifth year students. This finding may appear unexpected, as senior students usually have more clinical experience. However, it may reflect closer exposure of third and fourth year students to recent basic dental sciences such as microbiology, pharmacology, infection-control teaching, stricter supervision during earlier clinical training, or greater adherence to clinical instructions when students are still adapting to the clinical environment. In contrast, fifth year students may become more procedure oriented and may rely on routine habits, which could reduce attention to some infection control details. This pattern supports the need for repeated reinforcement of IPC concepts throughout all clinical years. The spiral curriculum model developed by cognitive psychologist Jerome Bruner; emphasizes that important topics should be revisited with increasing complexity over time, allowing earlier theoretical knowledge to be connected with later clinical application (31). Therefore, MRSA and other infection-control education should not be limited to early microbiology or introductory clinical courses, but should be repeatedly integrated into senior clinical training through case discussions, clinical audits, supervision, and feedback (32)

Gender was also significantly associated with infection-control practice score in the adjusted model, with female students scored about 4.1% points higher in infection-control practice than male students. This finding may reflect differences in perceived risk, preventive orientation, or self-reported adherence to clinical protocols. Gender differences in infection-control compliance have been discussed in the broader healthcare literature, where gender has been considered one of the factors that may influence compliance with infection-control precautions. However, findings across dental settings are not consistent. For example, a study from Riyadh, Saudi Arabia, found small differences between males and females in specific IPC practices such as hepatitis B vaccination and protective eyewear use (33). Similarly, a Jordanian study among dental, dental hygiene, and dental technology students found that gender was not a significant variable affecting infection-control practice (34). On the other hand, our result came compatible with results from studies particularly in nursing and dentistry, that demonstrate superior infection-control practices in female compared to their male peers, including better hand hygiene and compliance with safety protocols (35, 36). This difference may be partly explained by behavioral and social factors that influence risk perception, responsibility, and compliance with preventive measures. with research indicating that male students are associated with a higher risk of inadequate precaution compliance.

The selective association between GPA and practice may reflect the difference between academic achievement and clinical behavioral compliance. Students with a GPA of 3.0-3.49 had significantly higher practice scores than those with a GPA of 2.5-2.99, while the highest GPA category did not show a significant association. This pattern suggests that infection-control practice does not increase linearly with academic performance (37). This finding should not be interpreted as high-achieving students having poorer practice, but rather as compliance with health precautions is not always highest among the highest academic achievers, but is influenced by balanced academic performance, training, moral/professional responsibility, supervision, and clinical context. This interpretation is supported by Jeon et al, who reported that nursing students with moderate-to-high academic performance demonstrated greater adherence to standard precautions, suggesting that compliance may be influenced by balanced academic performance, professional responsibility, and readiness for clinical practice (38), rather than by academic excellence and theoretical achievement alone.

This study has some limitations that should be acknowledged. The cross-sectional design limits the ability to establish causal relationships between knowledge, training, and practice, despite higher knowledge and more frequent IPC training was associated with better practice. In addition, practice was measured using self-reported responses, which may be affected by recall bias and social desirability bias where students may over-report their compliance with infection-control procedures compared with their actual observed behavior. The study was also conducted in a single institution, which may limit the generalizability of the findings to other dental schools or healthcare education settings. Furthermore, the achieved sample size was below the initially calculated size, was predominantly female and included a larger proportion of fifth-year students, which may have influenced some subgroup comparisons and generalizability. Finally, although the regression model explained a substantial proportion of the variance in practice scores, other factors such as direct clinical supervision, availability of PPE, workload, clinic culture, and actual observed compliance were not directly measured. On the other hand, the study focused on MRSA-related knowledge, attitudes, and infection-control practices among clinical dental students, a group with direct patient contact and potential exposure to aerosol-generating procedures, provides useful local evidence from a dental education setting where infection-control training is highly relevant to patient safety, antimicrobial-resistance prevention, and dental student education. Second, the study assessed not only descriptive KAP levels but also the predictors of infection-control practice using adjusted linear regression. This allowed identification of educationally relevant factors, particularly knowledge score and IPC training frequency. Third, the questionnaire demonstrated acceptable internal consistency in the full analytic sample, supporting the reliability of the study measures.

## 5 Conclusion

Clinical dental students reported relatively high infection-control practice scores despite lower MRSA-specific knowledge levels. Higher knowledge score and more frequent IPC training were significant independent predictors of better practice, while year of study, gender, and GPA also showed significant associations in the adjusted model. These findings suggest that good infection-control practice is not merely routine compliance, but is supported by knowledge, repeated training, and educational exposure. Strengthening MRSA-related education and reinforcing IPC training throughout clinical dental educational years may improve students' preparedness for safe patient care and antimicrobial-resistance prevention.

## Data Availability

The raw data supporting the conclusions of this article will be made available by the authors, without undue reservation.
