# Supplementary material for: MRSA-related knowledge, attitudes, and practices among clinical dental students: a cross-sectional study with implications for infection prevention and control education and training
Source: Front Oral Health. 2026 Aug 31;7:1928942. doi: 10.3389/froh.2026.1928942 (PMC13572252; doi:10.3389/froh.2026.1928942)
Supplement: Supplementary file 1 [file Supplementaryfile1.docx]

**Supplementary Table 1. Item-level knowledge regarding MRSA**

| Knowledge item | Correct answer | Correct n | Correct % |
| --- | --- | --- | --- |
| Type of microorganism | Bacteria | 131 | 78.9 |
| Meaning of MRSA | Methicillin-resistant *Staphylococcus aureus* | 119 | 71.7 |
| Most common colonization site | Nasal cavity | 105 | 63.3 |
| Estimated colonization in general population | 1-2% | 37 | 22.3 |
| Population most likely to develop MRSA infection | Immunocompromised patients | 110 | 66.3 |
| Routes of transmission | All of the above | 108 | 65.1 |
| Colonization without symptoms | True | 97 | 58.4 |
| Possible MRSA infections | All of the above | 109 | 65.7 |
| Antibiotic misuse contributes to MRSA | True | 132 | 79.5 |
| HA-MRSA is generally less resistant than CA-MRSA | False | 66 | 39.8 |
| Antibiotic used for nasal colonization | Topical mupirocin | 45 | 27.1 |
| Survival on environmental surfaces | True | 107 | 64.5 |
| Standard sterilization can kill MRSA | True | 94 | 56.6 |
| Recommended equipment disinfection approach | Use MRSA-approved disinfectants | 131 | 78.9 |

**Supplementary Table 2. Item-level attitude regarding MRSA**

| Attitude item | Agreement % | Mean ± SD |
| --- | --- | --- |
| I feel confident in my knowledge about MRSA infection control | 24.7 | 2.91 ± 0.98 |
| Dental students should receive more education about MRSA prevention and control | 74.1 | 3.99 ± 1.10 |
| MRSA represents a potential risk in a dental clinic | 69.9 | 3.88 ± 0.74 |
| Dental healthcare personnel are at high risk of contracting MRSA | 63.9 | 3.67 ± 0.88 |
| MRSA colonization status should be checked before dental procedures | 40.3 | 3.22 ± 0.97 |
| Dental healthcare workers should be aware of MRSA colonization status and risks | 75.3 | 4.03 ± 0.97 |
| Dental practitioners play an important role in preventing MRSA transmission | 71.1 | 3.91 ± 0.92 |
| MRSA infection-control procedures are emphasized enough in dental curricula | 27.1 | 2.99 ± 0.89 |
| Unnecessary antibiotic prescriptions could contribute to MRSA | 59.7 | 3.75 ± 0.88 |
| Gloves and hand hygiene are enough to prevent MRSA spread | 18.7 | 3.32 ± 0.93 |
| Strict infection-control measures are essential | 65.6 | 3.95 ± 0.82 |
| PPE and instrument disinfection can reduce MRSA transmission | 66.9 | 3.82 ± 1.00 |

**Supplementary Table 3. Self-reported infection-control practices regarding MRSA**

| Practice item | Often/Always % | Mean ± SD |
| --- | --- | --- |
| Uses gloves when exposed to patient mucosa | 90.3 | 4.72 ± 0.77 |
| Performs hand hygiene before and after patient contact | 82.0 | 4.46 ± 0.85 |
| Changes gloves between patients | 90.4 | 4.77 ± 0.70 |
| Wears PPE during clinical procedures | 82.0 | 4.42 ± 0.91 |
| Performs hand hygiene before and after wearing gloves | 69.9 | 4.14 ± 1.08 |
| Disinfects dental unit surfaces between patients | 90.9 | 4.74 ± 0.70 |
| Follows standard infection-control guidelines | 88.6 | 4.61 ± 0.76 |
| Does not leave the work zone while wearing gloves | 83.2 | 4.41 ± 0.95 |
| Uses high-volume evacuator during aerosol-generating procedures | 60.3 | 3.81 ± 1.29 |
| Does not use phone while wearing gloves | 68.1 | 4.08 ± 1.16 |
| Checks sterilization indicators routinely | 65.0 | 4.00 ± 1.26 |

**Supplementary Table 4. Post-hoc comparisons for significant Kruskal-Wallis tests.**

| **Grouping variable** | **KAP domain** | **Comparison** | **Group A median (IQR)** | **Group B median (IQR)** | **Adjusted post-hoc p-value** | **Direction of difference** |
| --- | --- | --- | --- | --- | --- | --- |
| Year of study | Knowledge | Third year vs Fifth year | 11 (10-14) | 7 (5-10) | **<0.001** | Third year higher |
| Year of study | Knowledge | Third year vs Fourth year | 11 (10-14) | 8 (5-12) | **0.001** | Third year higher |
| Year of study | Attitude | Third year vs Fifth year | 46 (44-49) | 42 (36-46) | **<0.001** | Third year higher |
| Year of study | Attitude | Fourth year vs Fifth year | 46 (40-50) | 42 (36-46) | **0.011** | Fourth year higher |
| Year of study | Practice | Third year vs Fifth year | 54 (51-55) | 46 (41-50) | **<0.001** | Third year higher |
| Year of study | Practice | Fourth year vs Fifth year | 51 (46-54) | 46 (41-50) | **0.002** | Fourth year higher |
| Year of study | Practice | Third year vs Fourth year | 54 (51-55) | 51 (46-54) | **0.019** | Third year higher |
| Patient interaction frequency | Practice | Rarely (<2 patients/week) vs Occasionally (3-7 patients/week) | 54 (49-55) | 49 (44-53) | **<0.001** | Rarely higher |
| GPA range | Practice | 3.0-3.49 vs 3.5-4.0 | 51 (48-55) | 47 (44-51) | **0.039** | 3.0-3.49 higher |
| Source of knowledge | Knowledge | Course lecture/academic curriculum vs Internet/social media | 10 (8-13) | 6 (4-10) | **0.047** | Curriculum higher |
| Source of knowledge | Knowledge | Course lecture/academic curriculum vs Personal assumption | 10 (8-13) | 2 (2-3) | **<0.001** | Curriculum higher |
| Source of knowledge | Knowledge | Scientific articles/journals vs Personal assumption | 6 (4-10) | 2 (2-3) | **0.047** | Scientific articles/journals higher |
| Source of knowledge | Attitude | Course lecture/academic curriculum vs Personal assumption | 46 (42-49) | 36 (36-38) | **0.004** | Curriculum higher |
| Source of knowledge | Attitude | General knowledge vs Personal assumption | 45 (41-47) | 36 (36-38) | **0.024** | General knowledge higher |
| Source of knowledge | Practice | Course lecture/academic curriculum vs Internet/social media | 51 (48-55) | 38 (34-49) | **<0.001** | Curriculum higher |
| Previous IPC workshop/training frequency | Knowledge | Often vs Never | 9 (6-12) | 6 (2-7) | **0.020** | Often higher |
| Previous IPC workshop/training frequency | Knowledge | Always vs Never | 9 (7-12) | 6 (2-7) | **0.015** | Always higher |
| Previous IPC workshop/training frequency | Practice | Always vs Rarely | 54 (51-55) | 48 (44-54) | **0.004** | Always higher |
| Previous IPC workshop/training frequency | Practice | Always vs Never | 54 (51-55) | 45 (40-49) | **<0.001** | Always higher |
| Previous IPC workshop/training frequency | Practice | Always vs Often | 54 (51-55) | 50 (46-53) | **0.040** | Always higher |
| Previous IPC workshop/training frequency | Practice | Always vs Sometimes | 54 (51-55) | 47 (36-52) | **<0.001** | Always higher |
